# Hybrid Ensemble Model for Predicting the Strength of FRP Laminates Bonded to the Concrete

**DOI:** 10.3390/polym14173505

**Published:** 2022-08-26

**Authors:** Anas Abdulalem Alabdullh, Rahul Biswas, Jitendra Gudainiyan, Kaffayatullah Khan, Abdullah Hussain Bujbarah, Qasem Ahmed Alabdulwahab, Muhammad Nasir Amin, Mudassir Iqbal

**Affiliations:** 1 Department of Civil and Environmental Engineering, College of Engineering, King Faisal University, Al-Ahsa 31982, Saudi Arabia; 2 Department of Applied Mechanics, Visvesvaraya National Institute of Technology, Nagpur 440010, India; 3 Department of Civil Engineering, GLA University, Mathura 281406, India; 4 Department of Civil Engineering, University of Engineering and Technology, Peshawar 25120, Pakistan

**Keywords:** FRP, interfacial bond strength, concrete, hybrid model, performance analysis

## Abstract

The goal of this work was to use a hybrid ensemble machine learning approach to estimate the interfacial bond strength (IFB) of fibre-reinforced polymer laminates (FRPL) bonded to the concrete using the results of a single shear-lap test. A database comprising 136 data was used to train and validate six standalone machine learning models, namely, artificial neural network (ANN), extreme machine learning (ELM), the group method of data handling (GMDH), multivariate adaptive regression splines (MARS), least square-support vector machine (LSSVM), and Gaussian process regression (GPR). The hybrid ensemble (HENS) model was subsequently built, employing the combined and trained predicted outputs of the ANN, ELM, GMDH, MARS, LSSVM, and GPR models. In comparison with the standalone models employed in the current investigation, it was observed that the suggested HENS model generated superior predicted accuracy with R^2^ (training = 0.9783, testing = 0.9287), VAF (training = 97.83, testing = 92.87), RMSE (training = 0.0300, testing = 0.0613), and MAE (training = 0.0212, testing = 0.0443). Using the training and testing dataset to assess the predictive performance of all models for IFB prediction, it was discovered that the HENS model had the greatest predictive accuracy throughout both stages with an R^2^ of 0.9663. According to the findings of the experiments, the newly developed HENS model has a great deal of promise to be a fresh approach to deal with the overfitting problems of CML models and thus may be utilised to forecast the IFB of FRPL.

## 1. Introduction

Reinforced concrete structures are composite materials of concrete and reinforcement which are subjected to wear and tear throughout the structure’s life span [1]. This emphasises the need for proper maintenance and sustainability of RC structures. To address the self-strengthening factors in a structure’s construction process, many new technologies have been introduced and implemented [2]. Fibre-reinforced polymer (FRP) laminates bonded to the concrete prism comprise one of the retrofitting methods which facilitates an increase in the structural capacity performance of beams [3,4,5], columns [6,7], and beam–column joints [8,9,10,11]. The FRP plates exhibit outstanding resistance to corrosion [12], fatigue [13,14], creep, and hygrothermal deformations [15] and lead to lightweight and high-strength structures that can substitute the use of structural reinforcement. Different types of FRPs, namely CFRP, BFRP, and GFRP, are used in structural applications owing to their cost and benefits. Although CFRP is expensive, it has very good mechanical characteristics and corrosion, creep, and fatigue resistances. Contrarily, GFRP and BFRP have relatively weak mechanical characteristics and corrosion resistance, especially when exposed to the alkaline environment of concrete [16,17,18]. They are ductile, easy to install, and possess no magnetic properties, all of which are advantages of the FRP [19,20,21,22,23,24,25,26].

The reinforced concrete structure proceeds through different failure patterns. The cracks are possibly generated from the concrete, which leads to the failure of the concrete [27,28,29,30,31,32,33]. The FRP laminates are widely used for retrofitting reinforced concrete structures. The various failure patterns FRP is subjected to are rupture, concrete crushing, shear cracking, and delamination [34]. The rupture and crushing phenomenon in FRP occurs when the ends of the plates are tightly anchored. The FRP is commonly reported to fail due to debonding [35]. Debonding failure occurs in different conditions such as those of increasing temperature or the degradation of resin due to dynamic loading and thermal loading [36]. Irreversible interfacial debonding occurs due to the diffusion of water molecules in the plates and the interfacial bond failure is affected by bond quality due to workmanship [37]. The composite action between the concrete prism and FRP is responsible for the failure of strengthening members [38].

The flexural strengthening of beams can be achieved by increasing the efficiency of bonding or by providing a suitable interface between the concrete and FRP [39]. Most of the premature failures are due to inappropriate preparation of the interface between the concrete and FRP. Epoxy interlocking in near-surface mounting techniques can be used in such conditions. The smooth interface is generally extracted by the removal of the deteriorated surface layer of the concrete until the exposure of the coarse aggregates. The exposed surface of the concrete is concealed by sandblasting and removal of dust is carried out by using a special brush; additionally, the cleaning can be conducted with solvents and by drying before installing the FRP sheets. This essentially creates interfacial consistency and enhancement in the ultimate rupture strength by delayed debonding. The FRP can be used directly on concrete or laminates placed in the grooves and then packed using high-adhesive materials [38]. They can even be externally bonded to the grooves made on the concrete surface [40]. The Near Surface-Mounted Retrofit (NSMR) method is also widely used owing to its high resistance against environmental conditions [41,42]. Using epoxy mortars, the FRP bar is put into a slit in the cover concrete and joined to the concrete. Since FRP is not exposed on, it is beneficial in mitigating the aforementioned environmental effects even if it necessitates additional concrete slit formation work. The methods are considered according to the surface area, availability of materials and equipment, safety, and cost.

The single-lap shear test (SST) is an experimental procedure that can be conducted to calculate interfacial bond strength (IBS) between FRP and concrete prisms and its fundamental results are reliable and simple [43,44,45]. Many empirical or semi-empirical formulations have been conducted under the fundamental of IBS on experimental results from SSTs [29]. The empirical relations and the models generated seemed to correlate with the experimental data established. The basic simplified assumptions have been used in empirical relations, although the models have not been validated for new data. Artificial intelligence (AI) has commonly been used to obtain an optimistic solution for regression and classification of problems in engineering problems [46,47,48,49,50,51,52,53,54,55,56]. It can not only be used for experimental observations, but can also authenticate them by assigning new data sets [47,48,49,50,51,57,58,59,60,61,62,63,64,65,66,67,68,69,70,71,72,73,74,75,76].

Vu and Hoang [77] used the non-linear capabilities of the least square support vector machine to predict the punching shear capacity of FRP-reinforced concrete beams, which helped them to achieve the coefficient of determination (R^2^) equalling 0.99. Hong [78] used an artificial neural network (ANN) to determine the punching shear capacity of steel-fibre-reinforced concrete slabs. Abuodeh [79] studied the behavior of RC beams concerning their shear capacity under a neural interpretation diagram (NID) and recursive feature elimination (RFE) algorithm.

In the brief, we can state that AI models have proved to be very feasible and reliable for experimental results when it comes to the prediction of IBS of FRP plates or punching shear capacity of FRP-reinforced concrete beams. AI models such as multilinear regression, support vector machine, and ANN were used to forecast the IBS of laminates to the concrete prism by Su et al. [80]. The observation for the training and validation of the data resulted in the accuracy of R^2^ equalling 0.81 and 0.91, but the developed models can be developed based on their accuracy. We can also use gene expression programming (GEM), which is a robust technique used to form an intact relationship between input and output attributes, in the form of a simple mathematical equation. However, the ANN models provide no information about the basis of the relationship between the mathematical equations and attributes related to each other [81,82,83], and hence an overall parametric analysis should be conducted to investigate the effects of input attributes on IBS as they have an efficient command over the type of strengthening techniques in terms of efficiency and viability.

**Figure 1:**
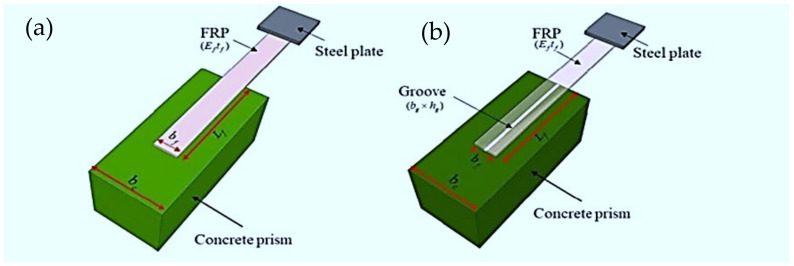
Single-lap shear test: (**a**) FRP externally bonded on concrete; (**b**) FRP externally bonded on the grooves of concrete (reprinted/adapted with permission from Su et al. [80]).

Taking these considerations into account, a hybrid ensemble approach was used to forecast ISB of FRP laminates externally bonded to the concrete prism on groves in this study. Using data from a custom database, we presented, analysed, and discussed six widely used conventional soft computing techniques: an artificial neural network (ANN) [84], extreme machine learning (ELM) [85], the group method of data handling (GMDH) [86], the multivariate adaptive regression splines (MARS) [87], the least squares-support vector machine (LSSVM) [81], and Gaussian process regression (GPR) [88]. When the conventional machine learning (CML) models were combined with an ANN model, a hybrid ensemble technique was proposed. For “unstable” models, an ensemble model works on a high level to improve their performance. It is worth noting that in many situations, an ensemble model comprised of multiple machine learning (ML) models outperforms a single model. The hybrid ensemble model (HENS) [89,90] presented in this paper comprised six CML models and one ANN. FRP laminates externally bonded on the concrete prism utilising SSTs results were predicted using conventional and HENS models, respectively, to aggregate the CML models’ output. Eight performance indices were used to evaluate the prediction performance of the models used, including ANN, ELM, GMDH, MARS, LSSVM, GPR, and HENS. The suggested HENS model’s benefits and applicability were confirmed by comparisons with CML models based on experimental data. The research plays an important role to explore the potential of the HENS model in the prediction of the ISB of FRP laminates externally bonded to the concrete prism on the groves using SST results (anchorage made on one end of FRP to the concrete prism shown in Figure 1). For better representation of the results, the study also includes the visual interpretation of results using an accuracy matrix and Taylor diagram.

## 2. Research Significance

In order to solve the challenges associated with using empirical formulas to forecast the IBS of FRP, it was clear that an alternative approach was required as soon as possible. For this purpose, the approaches of soft computing, with their proficiency for non-linear modelling, have emerged as a significant class of prediction tools, providing answers to the ever-increasing complexity of optimisation issues. In this research, an enhanced application of CML algorithms was presented for the estimation of IBS of FRP using a hybrid ensemble technique. The traditional feed-forward ANN was chosen for the construction of the HENS model because of its straightforward network topology, its capacity to deal with varying degrees of complexity, its ease of application, and its capability to provide highly non-linear modelling. The findings of this research will provide engineers with tools to predict the IBS of FRP and will make it easier to design FRP constructions that are less complex and more durable while also improving the level of accuracy with which such designs can be made.

## 3. Soft Computing Techniques

### 3.1. ANN

Artificial neurons [84] are conceptually developed from biological neurons, which are made up of elements that operate in parallel and are organised in ways that resemble biological neural networks. Each artificial neuron receives inputs and generates a single output that can be transmitted to a number of other neurons. There are three essential layers of ANN: the input layer, the output layer, and the hidden layer. The outputs of neurons are achieved by calculating the weighted total of all the inputs, which is then multiplied by the weights of the connections between the inputs and the neurons. Later, a bias term is added to this total. The weighted sum is processed through a (typically non-linear) activation function to create the output. The system generates a result that is similar to the target result. For this study, a single hidden layer was used to develop the model.

For $x\in R^{d}$, $with\;Kij\;\left(xi,\;xj\right)i,j=1,\ldots ,n$ for all pairs of $x\in R^{d}$ could make $K\left(X,X\right)$ the covariance matrix.

### 3.2. ELM

ELM is a novel soft computing approach that aims to bridge the gap between machine learning and biological learning mechanisms [85]. Extreme refers to a strong approach with learning capabilities comparable to the human brain in this context. Unlike ANNs, which require hidden neuron tuning during the learning phase, an ELM, whether it is a single- or multi-hidden layer feed-forward network, does not require any tuning. ELM has been effectively used in learning capacity for feature learning, clustering, regression, and classification applications in recent years [85,91]. ELM was created as a quick-learning, single-layer feed-forward network (SLFN) with high generalisation capacity, making it easier to build than other soft computing approaches.

In an ELM, the input data are mapped to an M-dimensional ELM random feature space and the network output is mapped according to the following equation:

(1) $$f_{M}\left(x\right)=h_{i}\left(x\right)$$

where $h_{i}$ = hidden nodes output for input *x*; M = dimensionality of the ELM random feature space; $\beta_{i}$ = output weight matrix between the hidden nodes and the output nodes.

### 3.3. GMDH

The group method of data handling (GMDH) is a set of techniques for computer-based structure identification and mathematical modelling [86]. The majority of GMDH algorithms employ an inductive self-organising method to generate a multi-parametric model. Inductive GMDH algorithms allow for the automatic discovery of data interrelationships, the selection of an ideal model or network architecture, and the improvement of the accuracy of current algorithms. The group method of data handling is a collection of methods for solving various issues. It includes methods for parametrics, clustering, analogue complexion, rebinarisation, and probability. This inductive technique is focused on sorting out more complex models and selecting the best solution with the minimum possible external criteria. Basic models include not just polynomials, but also non-linear, probabilistic functions and cauterisations. A GMDH model with several inputs and one output that is a subset of the base function’s components is written as:

(2) $$Y\left(x_{1},\ldots ,x_{n}\right)=a_{0}+\overset{m}{\underset{i=1}{\sum }}a_{i}f_{i}$$

where elementary functions’ dependency on different sets of inputs is denoted by $f_{i}$, $a_{i}$ are coefficients, and m represents the number of the base-function components.

GMDH methods use multiple component subsets of the base function (in eqn), termed as partial models, in order to obtain the optimal answer. The least squares approach is used to estimate the coefficients of these models. The number of partial model components in GMDH algorithms is continuously increased until the minimal value of an external criterion indicates a model structure with optimal complexity. This is referred to as model self-organisation.

### 3.4. MARS

**Figure 2:**
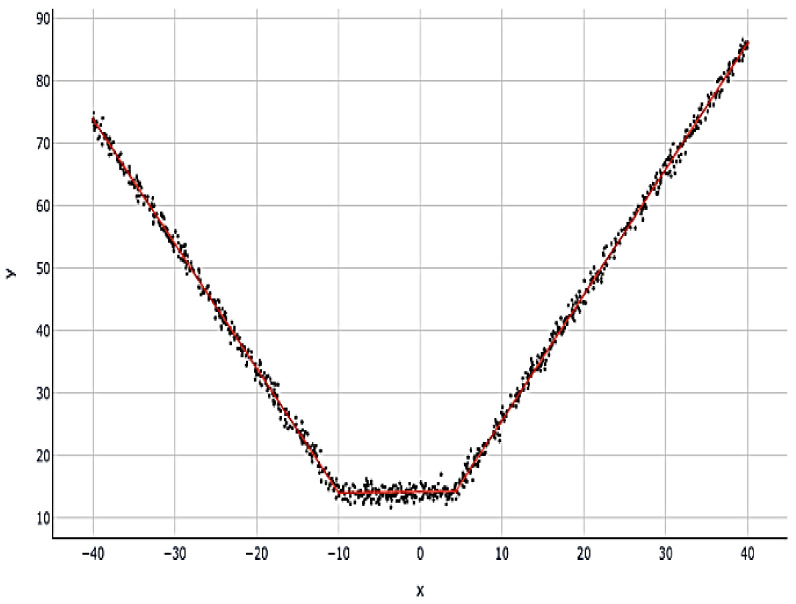
Example of a MARS model [62].

Jerome H. Friedman [87] proposed multivariate adaptive regression splines (MARS) as a type of regression model in 1991. It is a non-parametric regression approach, which may be seen as an extension of linear models, that incorporates nonlinearities and interactions between variables automatically. MARS may be thought of as an ensemble of linear functions linked together by one or more hinge functions. The results of mixing linear hinge functions may be seen in the figure below, where the black dots represent observations, and the red line is a MARS model forecast (Figure 2).

The algorithm is divided into two stages: forward and backward. In the forward step, it generates a large number of candidate basis functions in pairs. On the other hand, a pair of functions will be incorporated into the model if it lowers the overall model error. In the first phase, the maximum number of functions that the model generates with a hyper-parameter may be adjusted. Whereas, the pruning step, also known as the backward stage, goes over each function one by one and deletes those that do not contribute any value to the model. This is accomplished through the use of a cross-validation-based generalised cross-validation (GCV) score. It is only a rough estimate of the real cross-validation score, with the goal of penalising model complexity. A set of linear functions may be put forward as in the following simple equation:

(3) $$f\left(x\right)=\alpha_{0}+\overset{k}{\underset{i=1}{\sum }}\alpha_{i}B_{i}\left(x\right)$$

where, $f\left(x\right)$ is the output and $\overset{k}{\underset{i=1}{\sum }}\alpha_{i}B_{i}\left(x\right)$.Weighted sum of basis functions is denoted by $B_{i}\left(x\right)$. Each $\alpha_{i}c_{i}$ term represents a constant coefficient. 

### 3.5. LSSVM

A least-square support vector machine (LSSVM) is a supervised machine learning method that may be used for both regression and classification. It was created by Vapnik in 1995 [92] and is based on statistical learning theory. SVM techniques project data into a high-dimensional feature space and employ kernels to classify nonlinearly separable datasets [93,94]. In multidimensional space, an SVM model is essentially a representation of various classes in a hyperplane. SVM will generate the hyperplane in an iterative manner in order to reduce the error. SVM’s objective is to split datasets into classes such that a maximum marginal hyperplane (MMH) may be found. The data points closest to the hyperplane, or the points of a data set that, if deleted, would change the location of the dividing hyperplane, are called support vectors. As a result, they might be regarded as important components of data collection. In general, the accuracy of the SVR model is determined by the kernels used and their parameters. The radial basis function (RBF) has been shown to perform well as a kernel function for SVM in several forecasting experiments) [95].

For a data set $\omega =\left\{\left(x_{i},y_{i}\right)i=1,2\ldots n\right\}$ where $x\in R^{d}$ is a d-dimensional input vector space and y∈R is an output in a 1-dimensional vector space, SVM regression can estimate the relationship between *x* and *y*. In the SVM approach, the risk function is minimized by minimizing both empirical risk and $\|\omega {\|}^{2}$.

(4) $$R=\frac{1}{2}\|\omega {\|}^{2}+C_{C}\underset{}{\sum }l_{\varepsilon }(y_{i}-f\left(\underset{x_{i}}{\to }\right))$$

where the regression data vector is ‖ω‖ and the loss is denoted by $l_{\varepsilon }$, which presents the difference between $y_{i}$ (real output) and $f\left(\underset{x_{i}}{\to }\right)$. A positive constant value $C_{C}$ is needed to fix the abovementioned. $l_{\varepsilon }(y_{i}-f\left(\underset{x_{i}}{\to }\right))$ will be 0 for $y_{i}-f\left(\underset{x_{i}}{\to }\right)<\epsilon$. Otherwise, it is equal to $y_{i}-f\left(\underset{x_{i}}{\to }\right)\;$. The risk function can be minimized with the following function:

(5) $$f\left(x,\alpha ,\alpha^{*}\right)=\overset{l}{\underset{i=1}{\sum }}\left(\alpha_{i}^{*}-\alpha_{i}\right)\left(\varphi \left(x_{i}\right),\varphi \left(x\right)\right)+b$$

where $\alpha_{i}^{*}.\alpha_{i}=0$ and $\alpha_{i}^{*}.\alpha_{i}\geq 0$, $\varphi \left(x_{i}\right),\varphi \left(x\right)$ is a product of kernel function and *b* is a bias term.

### 3.6. GPR

Supervised learning has two key components, and their approaches are classification and regression issues. Gaussian process regression is one of the most appealing supervised learning nonparametric techniques for predictions among the many approaches [96]. Considering a data set:

(6) $$\omega =\;\left\{\left(x_{i},y_{i}\right)i=1,2\ldots n\right\}$$

where $x\in R^{d}$ is a d-dimensional input vector space and y∈R is an output in a 1-dimensional vector space, GPR regression can estimate the relationship between *x* and *y*. The conditional distribution of outcomes due to certain inputs is important for understanding the link between inputs and outputs in the regression technique. A joint Gaussian distribution covers a finite number of random variables in the Gaussian process. The Gaussian process *f*(*x*) can specify the mean and covariance functions:

$$\mathrm{Mean}\;\mathrm{function}\;m(x):\;m\left(x\right):m\left(x\right)=E\left[f\left(x\right)\right]$$

Covariance function or kernel function $K\left(x,x’\right):\;K\left(x,x’\right)=\left[\left(f\left(x\right)-m\left(x\right)\right)\left(f\left(x’\right)-m\left(x’\right)\right)\right]$.

## 4. Data Processing and Analysis

### 4.1. Descriptive Statistics and Statistical Analysis

**Figure 3:**
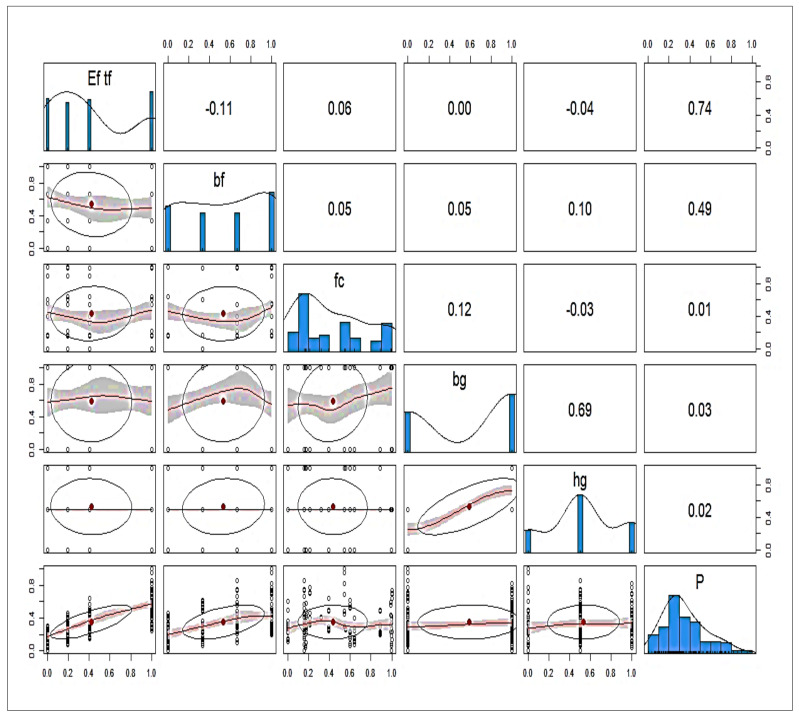
Pearson correlation with heat map.

Previous research yielded 136 experimental findings for a single lap-shear test [80], all of which were factored into the development of a HENS using CML. It is abundantly clear that the elastic modulus of FRP multiplied by the thickness of the fibre (*E_f_ t_f_*, GPa-mm), which is also known as the axial stiffness, the width of the FRP (*b_f_*, mm), the concrete compressive strength (*fc*, MPa), the width of the groove (*b_g_*, mm), and the depth of the groove (*h_g_*, mm) were all utilised as input variables, while the ultimate capacity (P, KN) was the desired variable. Table 1 presents the descriptive statistics of the input and output parameters. From this table, one can see that the *E_f_ t_f_* parameter ranged from 12.90 to 78.20 with a skewness of 0.58, the *b_f_* parameter ranged from 60 to 6270, the *b_g_* and *h_g_* parameter ranged from 10 to 1405, and the *fc* parameter ranged from 48.20 to 4585.40. However, the output value *p* ranged from 4.76 to 25.49 with a skewness of 0.80. After the descriptive analysis that was described above revealed that the collected database had a wide range of experimental data, statistical analysis was carried out in order to measure the degree of correlation (DOC) between the aforementioned parameters. This was carried out in order to draw the appropriate conclusions. According to the information presented by the Pearson Correlation in Figure 3, the DOC between *p* and other parameters (with the exception of *E_f_*, *t_f_*, and *b_f_*) was smaller when all of the parameters were examined. This is the conclusion reached by the information. On the other hand, it was shown that the DOC between *E_f_ t_f_* and *b_f_* was substantially higher.

### 4.2. Sensitivity Analysis

In general, sensitivity analysis (SA) is a technique that is used to determine how changes in input parameters affect the response of the proposed models. This will assist us in identifying the input parameters based on their influence on the result. The Cosine Amplitude Method [51] was used in this work to calculate the amount of influence of the inputs on the response, i.e., the IBS of FRP. The data pairings in this study were represented in a data array, *X*, as follows in Equation (7):

(7) $$X=\left\{x_{1},x_{2},x_{3},\ldots ,x_{i},\ldots ,x_{n}\right\}$$

and variable 𝑥𝑖 in 𝑋 is a length vector of 𝑚 as in Equation (8).

(8) $$x_{i}=\left\{x_{i1},xi_{2},x_{i3},\ldots ,x_{im}\right\}$$

The correlation between the strength of the relation (𝑅𝑖𝑗) and datasets of 𝑥𝑖 and 𝑥𝑗 are provided by Equation (9).

(9) $$R_{ij}=\frac{\sum_{k=1}^{m}x_{ik}x_{jk}}{\sqrt{\sum_{k=1}^{m}x_{ik}^{2}}\sum_{k=1}^{m}x_{jk}^{2}}$$

**Figure 4:**
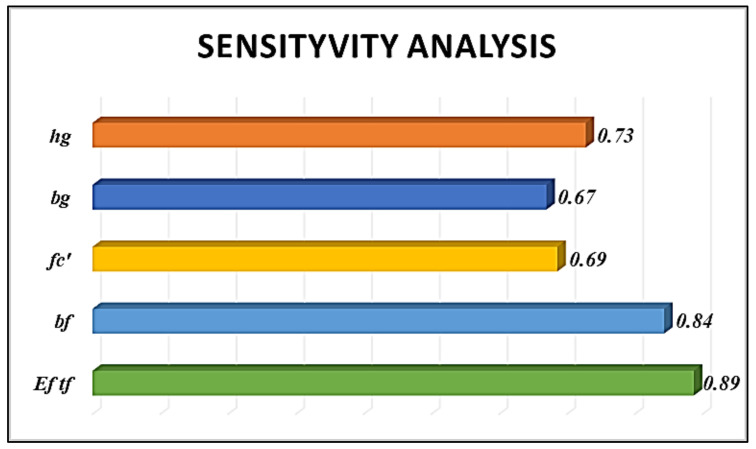
Sensitivity analysis of input parameters to output parameters.

The graphical representation of 𝑅_𝑖𝑗_ shows the relation between the IBS of FRP and the input parameters as shown in Figure 4. SA reveals that the *E_f_ t_f_* has the greatest influence on IBS with a strength value of 0.89 followed by *b_f_* and *h_g_* with strength values of 0.84 and 0.73 respectively. The parameters, *b_g_* and *fc* have strengths of about 0.67 and 0.69, respectively. It can be concluded that all the five parameters have higher influences on the interfacial bond strength and hence were considered in predicting the output.

### 4.3. Performance Parameters

To evaluate the effectiveness of the developed models, eight distinct performance indices (Equations (10)–(17) were calculated). These included the determination coefficient (R2), performance index (PI), variance account factor (VAF), Willmott’s index of agreement (WI), root mean square error (RMSE), mean absolute error (MAE), RMSE observation standard deviation ratio (RSR), and weighted mean absolute percentage error (WMAPE) [59,86,89,97,98,99,100,101,102,103,104,105,106,107,108,109,110,111,112,113,114,115,116,117,118,119]. The values of these indices should be identical to their ideal values, which are provided in Table 2. This will ensure that the prediction model is error-free. Take note that the ability of any predictive model to generalise is evaluated by determining various metrics, such as the degree of correlation, the associated error, the amount of variation, and so on, from these various aspects. This evaluation is carried out in order to determine the generalisation capacity of the model.


(10) $$R^{2}=\frac{\sum_{i=1}^{n}{\left(y_{i}-y_{mean}\right)}^{2}-\sum_{i=1}^{n}{\left(y_{i}-{\hat{y}}_{i}\right)}^{2}}{\sum_{i=1}^{n}{\left(y_{i}-y_{mean}\right)}^{2}}$$

(11) $$PI=adj.R^{2}+0.01VAF-RMSE$$

(12) $$VAF\;\left(\%\right)=\left(1-\frac{var(y_{i}-{\hat{y}}_{i})}{var(y_{i})}\right)\times 100$$

(13) $$WI=1-\;\left[\frac{\sum_{i=1}^{n}{\left(y_{i}-{\hat{y}}_{i}\right)}^{2}}{\sum_{i=1}^{n}{\left\{\left|{\hat{y}}_{i}-y_{mean}\right|+\;\left|y_{i}-y_{mean}\right|\;\right\}}^{2}}\right]$$

(14) $$RMSE=\sqrt{\frac{1}{n}\overset{n}{\underset{i=1}{\sum }}{\left(y_{i}-{\hat{y}}_{i}\right)}^{2}}$$

(15) $$MAE=\frac{1}{n}\overset{n}{\underset{i=1}{\sum }}\left|\left({\hat{y}}_{i}-y_{i}\right)\right|$$

(16) $$RSR=\frac{RMSE}{\sqrt{\frac{1}{n}\sum_{i=1}^{n}{\left(y_{i}-y_{mean}\right)}^{2}}}$$

(17) $$WMAPE=\frac{\sum_{i=1}^{n}\left|\frac{y_{i}-{\hat{y}}_{i}}{y_{i}}\right|\times y_{i}}{\sum_{i=1}^{n}y_{i}}$$

## 5. Results and Discussion

### 5.1. Simulation of Soft Computing Models

**Figure 5:**
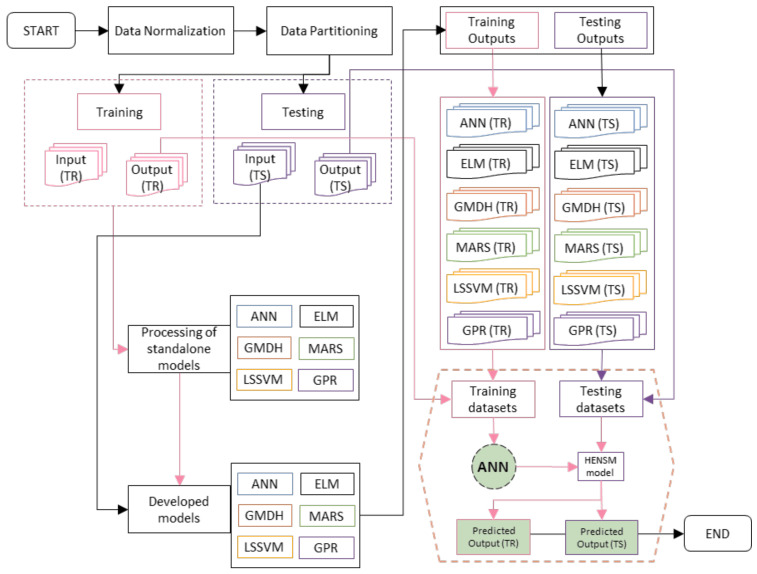
Flowchart of the implementation approach of the HENS Model.

By analysing 136 tests, we hoped to be able to forecast FRP’s interfacial bond strength. The goal of this research was to determine how the five input parameters affect the IBS using the most efficient CML models. After that, the outputs of the created CML models were aggregated using the ANN and the HENS model was built. To train the models, 70% of the dataset (i.e., the training dataset of 110 instances) was used, while the remaining data (28 instances) were used for testing. The prediction success was evaluated using eight performance parameters. With these indices, we could assess how well our models worked by comparing the actual and anticipated values and measuring correlation coefficients, variances, and related errors. The created models and their outputs are presented in the following subsections for a more in-depth comparison. The flowchart of developed models has been shown in Figure 5.

#### 5.1.1. ANN Model

**Figure 6:**
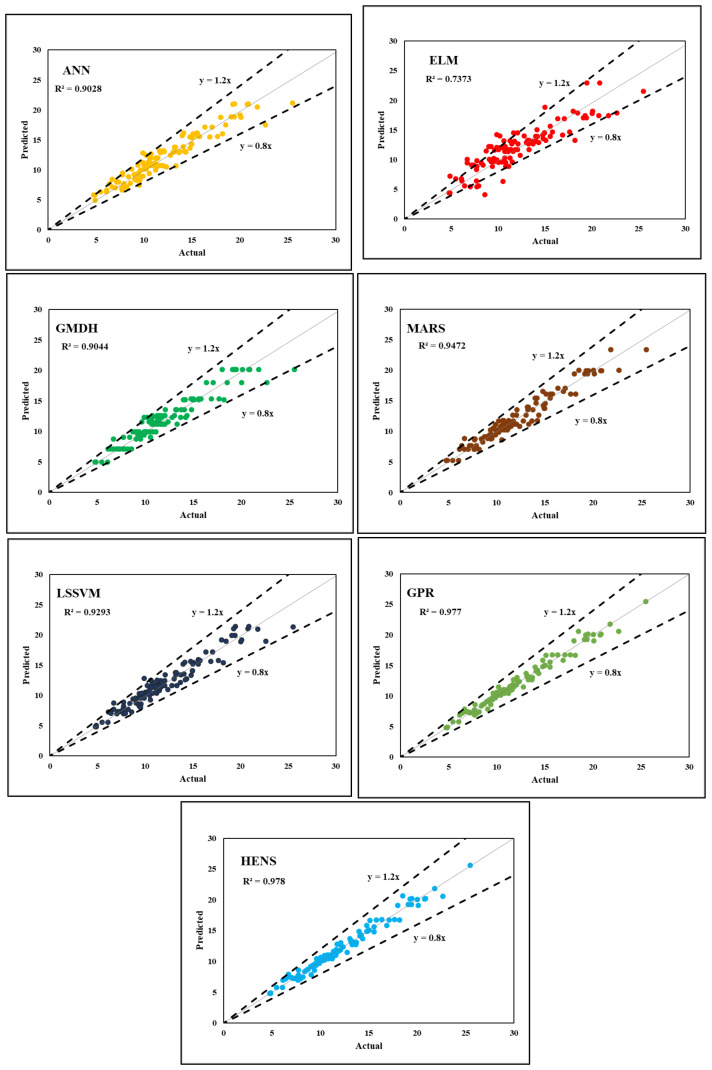
Tested vs. predicted graph of training data.

**Figure 7:**
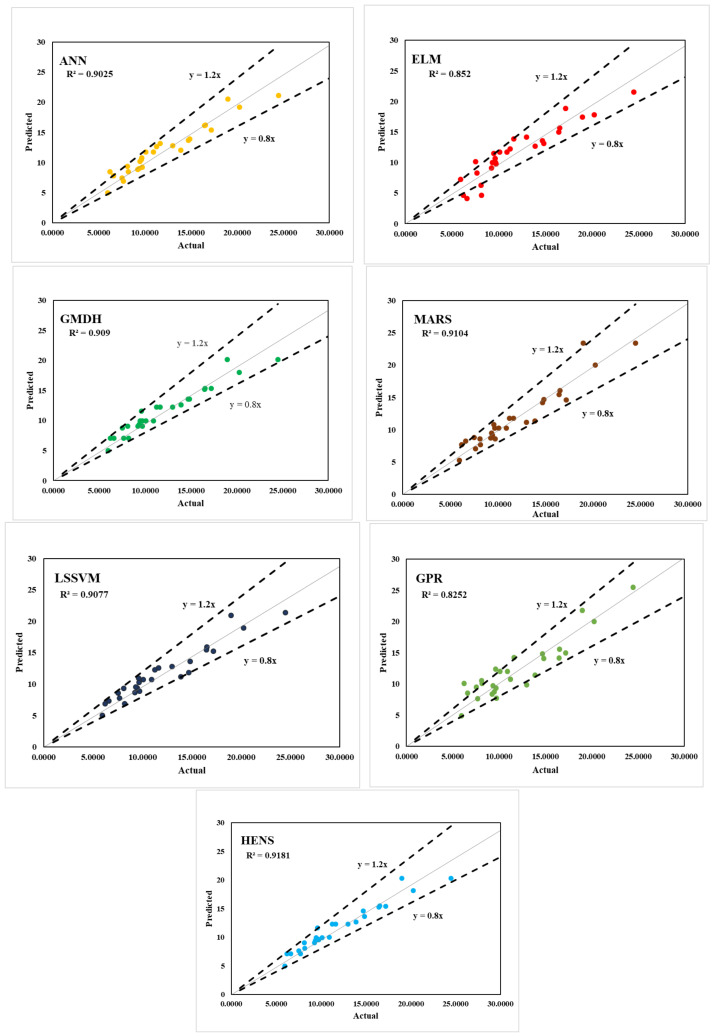
Tested vs. predicted graph of testing data.

It was determined that only five hidden layers were necessary, and the best model was found via trial and error. For example, Table 3 provides a breakdown of the model’s performance parameters. When ANN was being trained, it had an accuracy rate of over 91% (R^2^ = 0.9159), whereas when it was being tested, its accuracy rate increased to 92% (R^2^ = 0.9290). In terms of MAE (training = 0.0448 and testing = 0.0514) and RMSE (training = 0.0594 and testing = 0.0620), the best training results for the best feed-forward ANN structure were adopted with five inputs. For the training and testing datasets, the experimental and expected values of the ANN model are displayed in Figure 6 and Figure 7.

#### 5.1.2. ELM Model

In contrast, in order to figure out the structure of the ELM, the number of hidden neurons, which can range anywhere from 2 to 30, was counted. In this particular research project, the sigmoid activation function was put to use in order to choose the most effective ELM model. The optimal number of hidden neurons was determined to be six, which was arrived at through a process of trial and error. The model performance is tabulated in the Table 3 and Table 4. It was observed that the ELM model attained the R^2^ value of 0.7881 in training, whereas it improved little to the value of 0.8647 in the testing phase (Table 3 and Table 4).

#### 5.1.3. GMDH

The best-performing GMDH model was determined by a trial-and-error method with a maximum of five layers of neurons. The value of alpha was set to 0.6 to achieve the best result. When GMDH was being trained, it had an accuracy rate of over 91% (R^2^ = 0.9154), whereas when it was being tested, its accuracy rate increased to 93% (R^2^ = 0.9359). Table 3 and Table 4 provide a breakdown of the model’s performance parameters.

#### 5.1.4. MARS Modelling

Initial parameters, such as the maximum number of BFs, GCV penalty per knot, maximum number of interactions, and so on, need to be tuned properly in MARS modelling in order to produce the best predictive model, as was explained in the methodology section (refer to Section 3.4). Table 5 contains the final values of the effective parameters, which may be found by following this process.

For the purpose of constructing the MARS model, which is used for forecasting the desired output, piecewise linear combinations of BFs were employed in this method. In order to arrive at an accurate calculation of the IFB of FRP, each of the five variables were first analysed under a variety of scenarios, and then the ‘states’ were formulated. In the beginning, the MARS will build a number of linear combinations (also known as linear BFs), and then, at the end, it will produce a model (MARS) to predict the desired output (y). The specifics of the BFs that make up the MARS model are provided in Table 5, and the equation that represents the final model may be seen below (17).

The expression that is presented in Equation (17) can be utilised as a ready-made solution for problems that require a more realistic approach. FRP’s IFB may be calculated with an accuracy of R2 (training = 0.8941, testing = 0.8816), RMSE (training = 0.0675, testing = 0.0722), and MAE (training = 0.0499, testing = 0.0541). Table 6 presents the other performance characteristics in order to facilitate a detailed evaluation of the model.

(18) $$\begin{array}{r} y=0.31222\;+0.41602*BF1\;-0.61344*BF2\;+0.20701*BF3\\ -0.25568*BF4\;+0.33178*BF5\;-0.50944*BF6\\ -0.32458*BF7\;-0.39979*BF8\;-5.3741*BF9\\ +4.692*BF10\;-0.9563*BF11\;+18.166*BF\; \end{array}$$

#### 5.1.5. LSSVM Model

The performance of LSSVM depends on two parameters i.e., Sigma and gamma. For the optimum modelling by trial and error, the values of those parameters were found as 60.38 and 6656.09, respectively. The elapsed time for the execution of the model was found to be 0.0016 s. The best performing LSSVM yielded an R^2^ of 0.9346 in training with a value of WMAPE of 0.1107, whereas for testing the values were 0.9226 and 0.1523 respectively.

#### 5.1.6. GPR Model

Two critical parameters that can be found through an initial round of trial-and-error are S (width of rbf) and ε (gaussian noise), both of which are discussed in the technique section (refer to Section 3.6). S = 0.50 and ε = 0.01 can be used as design settings for GPR’s parameters. IBF values were predicted almost accurately by the GPR model during training, but a substantial variance was detected during testing. Figure 6 and Figure 7 show a scatter plot comparison of the actual and projected values for the training and testing datasets. Generalisation capability was demonstrated by the GPR model’s prediction accuracy (R^2^ = 0.9775; RMSE = 0.0306 in training, R^2^ = 0.8404; RMSE = 0.0944) in both stages. Table 3 and Table 4 provide further information on the various performance metrics.

#### 5.1.7. HENS Modelling

The results of six separate CML models were reported earlier in the section. As indicated in Table 3, all of the generated models were able to accurately estimate the IFB of FRP. Given the huge size of the testing dataset, the comparatively strong R^2^ (ranging from 0.7811 to 0.9775) and low RMSE (ranging from 0.0306 to 0.0938) prediction values show this (110 instances). However, a close examination of the data shows that GPR was the best standalone model, whereas ELM was the poorest. Other performance indices, such the WMAPE and PI, support GPR’s prediction performance at all levels, in contrast to the R^2^ and RMSE values. Furthermore, it is important to remember that a model that performs well in testing and has a high level of accuracy is generally considered to be a robust model. As a result, improving their validation performance may make it possible to construct an even more robust model. As a result, the ensemble technique was used in this work without any additional ML models being included. Once these issues are resolved, this will be a more positive outcome for everyone. Without enough data, the computer models produced a wide range of outputs with variable degrees of accuracy. When multiple models are combined, there is a lower chance of selecting the erroneous one. In this study, a HENS model was utilised to combine the six CML models used to forecast the IFB of FRP in an attempt to apply ensemble modelling. The standard feed-forward ANN was used to build the suggested HENS model, which aggregates the outputs of all six CML models as inputs and outputs the actual IFB value, as mentioned in the methodology section (refer to Section 3). Figure 5 depicts the HENS model’s implementation approach in the form of a flowchart, which makes it easy to see how amalgamation works. Table 3, Table 4, Table 5 and Table 6 show the HENS model’s ability to accurately anticipate outcomes. When compared with the solo CML models that had been created, the ensemble hybrid model yielded the best predictions. Table 3’s CML models were compared, and the HENS model achieved substantial results in all phases, R^2^ (training = 0.9783, testing = 0.9287), VAF (training = 97.83, testing = 92.87), RMSE (training = 0.0300, testing = 0.0613), and MAE (training = 0.0212, testing = 0.0443)) when compared. The predictability of the constructed model, which includes HENSM, was assessed using R^2^, VAF, RMSE, MAE, and other performance criteria. Staggered plots show how projected and actual values compare for training and testing datasets, respectively, in Figure 6 and Figure 7. The suggested HENS model is thoroughly evaluated in the following sections to ensure its robustness and generalisability.

### 5.2. Taylor Diagram

**Figure 8:**
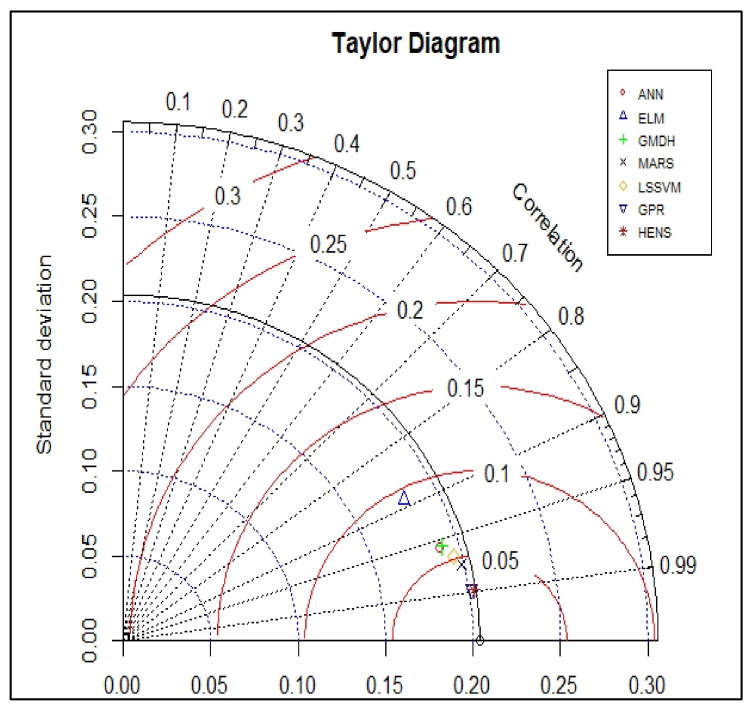
Taylor diagram of the training data.

**Figure 9:**
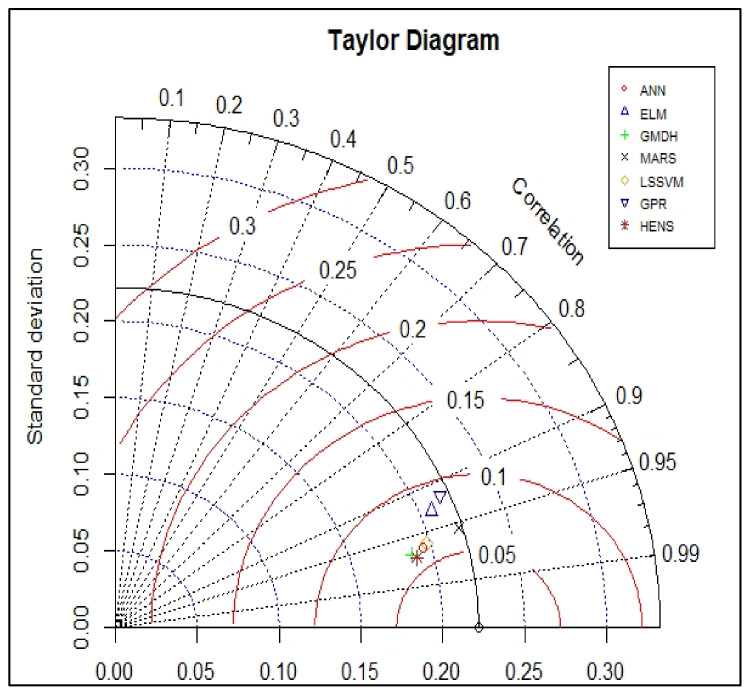
Taylor diagram of the testing data.

As shown in Figure 8 and Figure 9, the Taylor diagram was utilised to analyse the performance of the hybrid support vector machine models for testing and training datasets respectively. This diagram establishes whether or not the model is able to accurately forecast the intended result. In order to obtain a relative measure of how well the three models compare to one another, we looked at three distinct statistical criteria (RMSE, correlation coefficients, and standard deviation ratios). As the reference point, we use the centred RMSE, which is defined as the distance from the measured point. In the reference model, both the standard deviation and the correlation coefficient were both configured to have a value of 1. It is clear from looking at the graph that the values of the standard deviation and correlation coefficient for all seven models during the training phase were quite near to 1. It is possible to draw the conclusion from the graph that the ELM model had the lowest correlation in training, whereas the GPR and HENS model delivered the best performance during the training phase. Out of all five models, the HENS model had the best performance for the testing dataset. As a result, it is possible to draw the conclusion that the HENS model has the best overall performance because it produced satisfactory results for both datasets.

### 5.3. Accuracy Matrix

**Figure 10:**
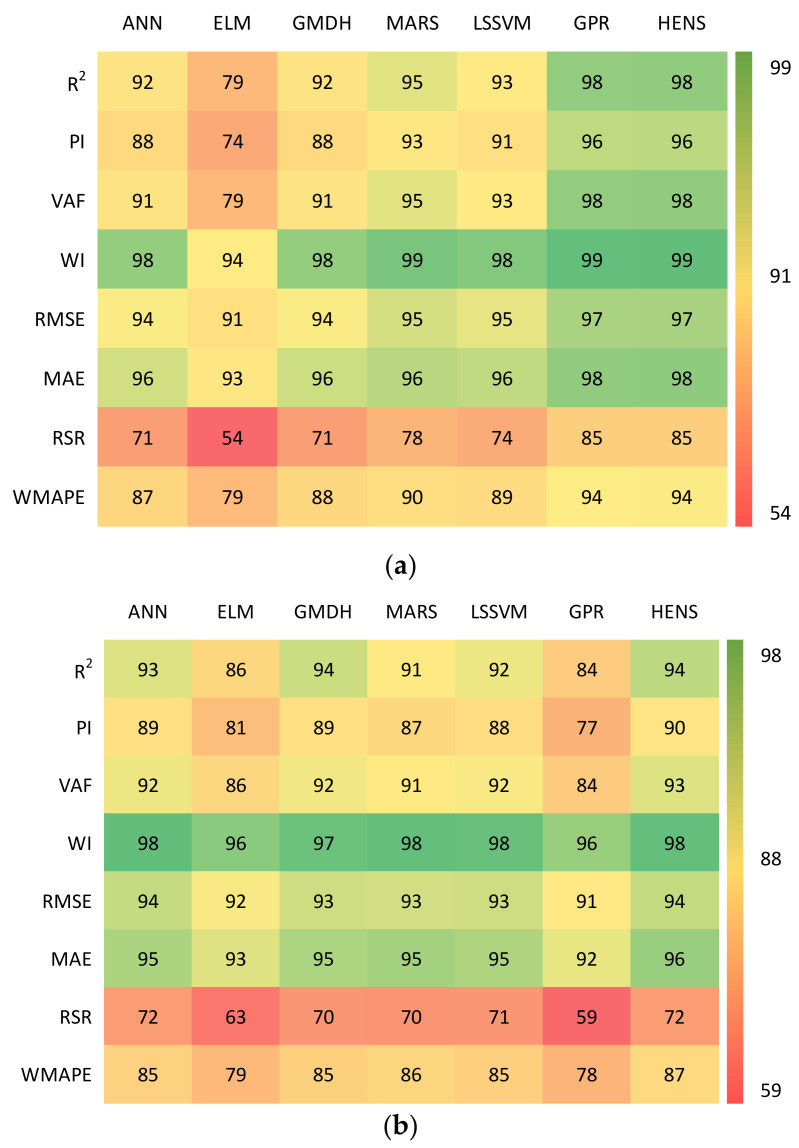

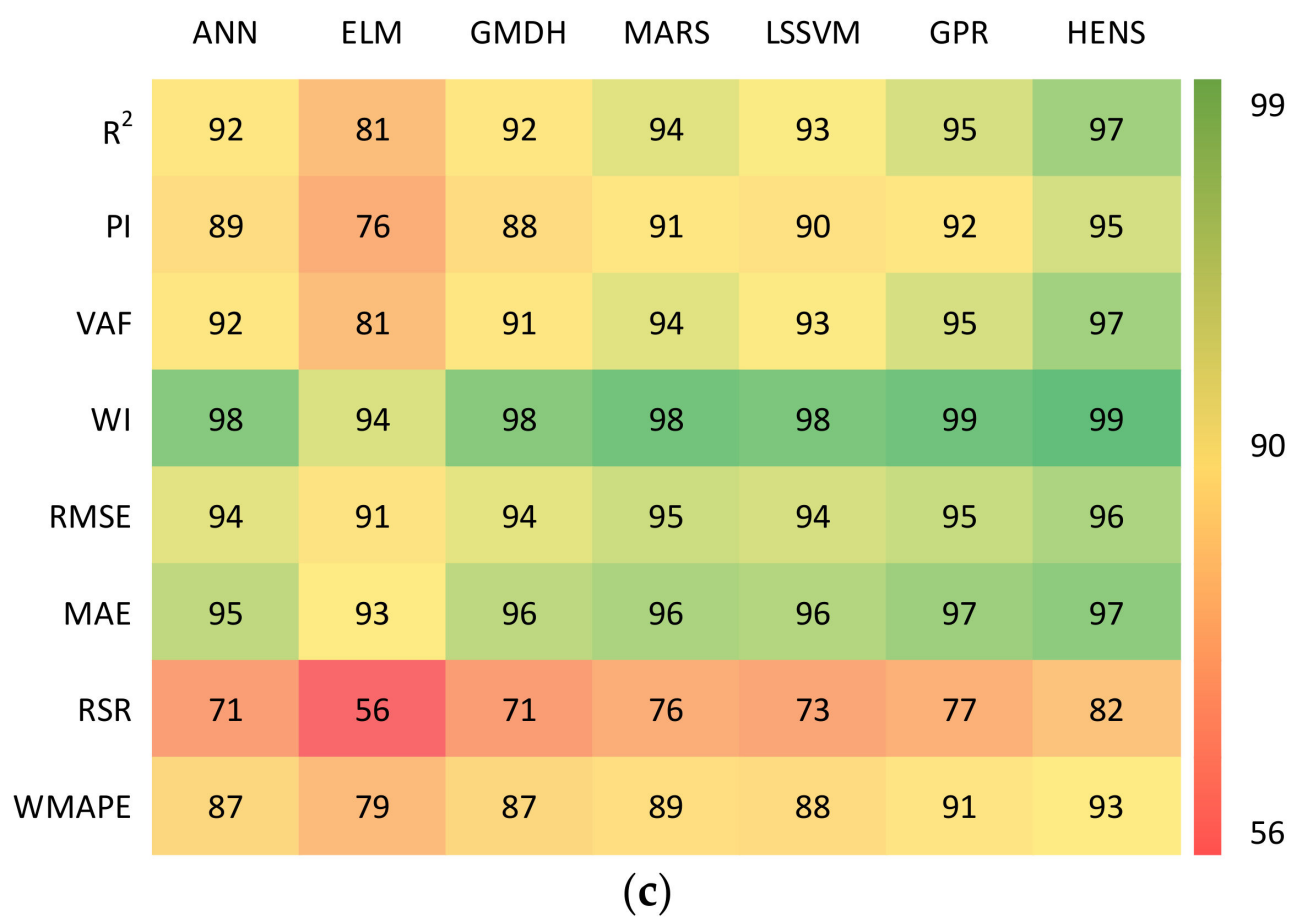
Accuracy matrix for (**a**) training, (**b**) testing, and (**c**) total datasets.

To better demonstrate the values of performance indices, a recently proposed heat map-shaped graphical assessment, called accuracy matrix, was tested to visualise the model efficiency [120]. This matrix displays multiple statistical parameters to measure the model’s predictive performance for the training and testing datasets. Figure 10 displays the accuracy matrix for the performance indices determined in this study. It indicates the accuracy of the performance parameters (in percentage) by comparing them with their corresponding ideal values. For example, the ideal value of MAE is 0 and, in the present work, the value of MAE for the training subset was determined to be 0.0448 for the ANN model (see Table 3. Thus, it can be estimated that the ANN model attained 96% ((1 − 0.0448) × 100%) accuracy in terms of MAE. On the other hand, the values of R2 and PI were obtained as 0.9421 and 1.7957 in the testing phase, respectively, for HENS (see Table 4), which shows that HENS attained 94% ((0.9421/1) × 100%) and 90% ((1.7957/2) × 100%) accuracy in terms of R^2^ and PI, respectively. A similar procedure was followed for the other parameters as well. However, it may be noted that the parameters such as VAF, which are determined in percentage terms, should be converted in their decimal form before implementing the abovementioned procedure.

## 6. Conclusions

It is important to note that an accurate and trustworthy estimate of the concrete IFB of FRP laminates can aid with cost/performance optimisation while also saving time. The estimation of IFB of FRPL was reported in the current work. In this work, six independent machine learning models—ANN, ELM, GMDH, MARS, LSSVM, GPR, and a HENS model—were introduced. A trustworthy experimental database made up of 136 FRPs was employed for this purpose. To train and validate the used models, the obtained dataset was split into training and testing subsets. Several statistical techniques were used to compare and analyse the accuracy of all models. Through the study, the following conclusions were drawn:

1. A conventional model, GPR provided the best result in training (R^2^ = 0.9775, VAF = 97.74, RMSE = 0.0306, and RSR = 0.15) whereas the GMDH model provided the best result in testing (R^2^ = 0.9359, VAF = 92.08, RMSE = 0.0655, and RSR = 0.2953).
2. It was observed from the experimental data that the suggested HENS model achieved the maximum prediction accuracy by minimising the particular flaws of CML models. Additionally, it is clear that the current HENS model (R^2^ = 0.9663, VAF = 96.60, RMSE = 0.0383, and RSR = 0.1847) was the best-performing model among the other models, according to Table 6, was able to handle the overfitting problem of the GPR model and exhibited all the desired trends in the parametric study of FRP, confirming the superiority of the suggested method at all levels.
3. The HENS model had high potential to forecast the intended IFB of FRPL bonded to concrete, as shown by the parametric analysis, and was extremely easy to implement. It also has a very cheap computational cost (only 10 s), is representational, and performed better than the standalone models.

In conclusion, this study successfully used an ensemble ML model to predict the IFB of FRPL bonded to concrete. It seems to be a precise and yet computationally effective technique. However, a thorough analysis of the outcomes suggests that the suggested strategy could be further enhanced in the future, possibly including a thorough evaluation of the HENS model approach for complicated issues in many technical and scientific domains. As far as the authors are aware, this study is the first to apply a hybrid ensemble of CML models to forecast IFB or FRPL bonded to concrete.

## Figures and Tables

**Table 1:** Descriptive statistics of the collected dataset [80].

Descriptive Statistic	Input Variables					Target Variable
	Elastic Modulus of FRP *x* Thickness of FRP, *E_f_ t_f_*	Width of FRP, *b_f_*	Concrete Compressive Strength, *f_c_*	Width of Groove, *b_g_*	Depth of Groove, *h_g_*	Ultimate Capacity, *p*
Unit	GPa-mm	mm	MPa	mm	mm	KN
Mean	40.33	46.10	33.72	7.94	10.33	12.05
Standard Error	2.18	1.01	0.73	0.21	0.30	0.37
Median	39.10	50.00	32.70	10.00	10.00	11.11
Mode	78.20	60.00	26.70	10.00	10.00	9.87
Standard Deviation	25.41	11.81	8.49	2.47	3.45	4.32
Sample Variance	645.42	139.52	72.15	6.10	11.93	18.65
Kurtosis	−1.23	−1.49	−1.11	−1.90	−0.88	0.30
Skewness	0.58	−0.13	0.49	−0.36	−0.09	0.80
Range	65.30	30.00	25.50	5.00	10.00	20.73
Minimum	12.90	30.00	22.70	5.00	5.00	4.76
Maximum	78.20	60.00	48.20	10.00	15.00	25.49
Sum	5484.80	6270.00	4585.40	1080.00	1405.00	1638.72
Count	136.00	136.00	136.00	136.00	136.00	136.00
Confidence Level (95.0%)	4.31	2.00	1.44	0.42	0.59	0.73

**Table 2:** Ideal values of different performance parameters.

Indices	R^2^	PI	VAF	WI	RMSE	MAE	RSR	WMAPE
Ideal Value	1	2	100	1	0	0	0	0

where y and $\hat{y}$ are the actual and estimated output; n is the total number of observations; and $y_{mean}$ is the average of the actual values.

**Table 3:** Performance indices for the training dataset.

Indices	ANN	ELM	GMDH	MARS	LSSVM	GPR	HENS
R^2^	0.9159	0.7881	0.9154	0.9496	0.9346	0.9775	0.9783
PI	1.7674	1.4722	1.7668	1.8509	1.8139	1.9233	1.9256
VAF	91.4997	78.8134	91.4947	94.9583	93.4556	97.7493	97.8314
WI	0.9767	0.9376	0.9768	0.9869	0.9827	0.9943	0.9945
RMSE	0.0594	0.0938	0.0595	0.0458	0.0521	0.0306	0.0300
MAE	0.0448	0.0735	0.0431	0.0357	0.0391	0.0214	0.0212
RSR	0.2916	0.4603	0.2917	0.2245	0.2558	0.1500	0.1474
WMAPE	0.1254	0.2076	0.1217	0.1004	0.1107	0.0604	0.0601

**Table 4:** Performance indices for the testing dataset.

Indices	ANN	ELM	GMDH	MARS	LSSVM	GPR	HENS
R^2^	0.9290	0.8647	0.9359	0.9121	0.9226	0.8404	0.9421
PI	1.7721	1.6148	1.7759	1.7355	1.7566	1.5489	1.7957
VAF	92.1975	86.4653	92.0816	91.0821	91.7866	83.7030	92.8774
WI	0.9776	0.9620	0.9740	0.9769	0.9753	0.9560	0.9775
RMSE	0.0620	0.0823	0.0655	0.0665	0.0655	0.0904	0.0613
MAE	0.0514	0.0716	0.0519	0.0482	0.0526	0.0757	0.0443
RSR	0.2794	0.3708	0.2953	0.2996	0.2950	0.4076	0.2764
WMAPE	0.1489	0.2075	0.1505	0.1395	0.1523	0.2192	0.1284

**Table 5:** Basis function of the MARS model.

Basis Function	Models
BF1	max (0, *E_f_ t_f_* − 0.18989)
BF2	max (0, 0.18989 − *E_f_ t_f_*)
BF3	max (0, *b_f_* − 0.66667)
BF4	max (0, 0.66667 − *b_f_*)
BF5	BF1 ×max (0, *b_f_* − 0.33333)
BF6	BF1 × max (0, 0.33333 − *b_f_*)
BF7	BF3 × max (0, *f_c_* − 0.39216)
BF8	BF1 × max (0, *f_c_* − 0.54118)
BF9	BF1 × max (0, 0.54118 − *f_c_*)
BF10	BF1 × max (0, 0.59608 − *f_c_*)
BF11	BF3 × max (0, *E_f_ t_f_* − 0.40123)
BF12	max (0, *f_c_* − 0.98824) × max (0, *E_f_ t_f_* + 0)

**Table 6:** Performance indices for the total dataset.

Indices	ANN	ELM	GMDH	MARS	LSSVM	GPR	HENS
R^2^	0.9184	0.8052	0.9167	0.9408	0.9303	0.9452	0.9663
PI	1.7719	1.5112	1.7677	1.8286	1.8025	1.8395	1.8927
VAF	91.6594	80.5153	91.4986	94.0692	92.9899	94.5057	96.6002
WI	0.9769	0.9435	0.9763	0.9846	0.9811	0.9859	0.9911
RMSE	0.0599	0.0917	0.0607	0.0506	0.0550	0.0487	0.0383
MAE	0.0461	0.0731	0.0448	0.0382	0.0418	0.0321	0.0258
RSR	0.2888	0.4415	0.2924	0.2436	0.2652	0.2347	0.1847
WMAPE	0.1300	0.2076	0.1273	0.1080	0.1188	0.0914	0.0734

## Data Availability

The data used in this research have been properly cited and reported in the main text.

## References

[1] Liberati E.A.P., Nogueira C.G., Leonel E.D., Chateauneuf A. (2014). Nonlinear formulation based on FEM, Mazars damage criterion and Fick’s law applied to failure assessment of reinforced concrete structures subjected to chloride ingress and reinforcements corrosion. Eng. Fail. Anal..

[2] Siddika A., Mamun M.A., Ferdous W., Alyousef R. (2020). Performances, challenges and opportunities in strengthening reinforced concrete structures by using FRPs–A state-of-the-art review. Eng. Fail. Anal..

[3] Yang J., Haghani R., Blanksvärd T., Lundgren K. (2021). Experimental study of FRP-strengthened concrete beams with corroded reinforcement. Constr. Build. Mater..

[4] Panahi M., Zareei S.A., Izadi A. (2021). Flexural strengthening of reinforced concrete beams through externally bonded FRP sheets and near surface mounted FRP bars. Case Stud. Constr. Mater..

[5] Kotynia R., Oller E., Marí A., Kaszubska M. (2021). Efficiency of shear strengthening of RC beams with externally bonded FRP materials –State-of-the-art in the experimental tests. Compos. Struct..

[6] Abedini M., Zhang C. (2021). Dynamic performance of concrete columns retrofitted with FRP using segment pressure technique. Compos. Struct..

[7] Hadi M.N.S. (2007). Behaviour of FRP strengthened concrete columns under eccentric compression loading. Compos. Struct..

[8] Tafsirojjaman T., Fawzia S., Thambiratnam D.P., Zhao X.L. (2021). FRP strengthened SHS beam-column connection under monotonic and large-deformation cyclic loading. Thin-Walled Struct..

[9] Lee W.T., Chiou Y.J., Shih M.H. (2010). Reinforced concrete beam-column joint strengthened with carbon fiber reinforced polymer. Compos. Struct..

[10] Wu Y.-F., Jiang C. (2013). Quantification of Bond-Slip Relationship for Externally Bonded FRP-to-Concrete Joints. J. Compos. Constr..

[11] Fathelbab F.A., Ramadan M.S., Al-Tantawy A. (2014). Strengthening of RC bridge slabs using CFRP sheets. Alex. Eng. J..

[12] Xian G., Guo R., Li C. (2022). Combined effects of sustained bending loading, water immersion and fiber hybrid mode on the mechanical properties of carbon/glass fiber reinforced polymer composite. Compos. Struct..

[13] Ding J., Cheng L., Chen X., Chen C., Liu K. (2021). A review on ultra-high cycle fatigue of CFRP. Compos. Struct..

[14] Kishore K. (2019). Experimental Analysis on Comparison of Compressive Strength Prepared with Steel Tin Cans and Steel Fibre. Int. J. Res. Appl. Sci. Eng. Technol..

[15] Guo R., Xian G., Li F., Li C., Hong B. (2022). Hygrothermal resistance of pultruded carbon, glass and carbon/glass hybrid fiber reinforced epoxy composites. Constr. Build. Mater..

[16] Li C., Yin X., Liu Y., Guo R., Xian G. (2020). Long-term service evaluation of a pultruded carbon/glass hybrid rod exposed to elevated temperature, hydraulic pressure and fatigue load coupling. Int. J. Fatigue.

[17] Wang Z., Zhao X.L., Xian G., Wu G., Singh Raman R.K., Al-Saadi S. (2017). Durability study on interlaminar shear behaviour of basalt-, glass- and carbon-fibre reinforced polymer (B/G/CFRP) bars in seawater sea sand concrete environment. Constr. Build. Mater..

[18] Li C., Guo R., Xian G., Li H. (2020). Innovative compound-type anchorage system for a large-diameter pultruded carbon/glass hybrid rod for bridge cable. Mater. Struct..

[19] Nagar P.A., Gupta N., Kishore K., Parashar A.K. (2021). Coupled effect of B. Sphaericus bacteria and calcined clay mineral on OPC concrete. Mater. Today Proc..

[20] Sharma P., Sharma N., Singh P., Verma M., Parihar H.S. (2020). Examine the effect of setting time and compressive strength of cement mortar paste using iminodiacetic acid. Mater. Today Proc..

[21] Jiang C., Wu Y.F. (2020). Axial strength of eccentrically loaded FRP-confined short concrete columns. Polymers.

[22] Bai Y., Nardi D.C., Zhou X., Picón R.A., Flórez-López J. (2021). A new comprehensive model of damage for flexural subassemblies prone to fatigue. Comput. Struct..

[23] Zhou X., Bai Y., Nardi D.C., Wang Y., Wang Y., Liu Z., Picón R.A., Flórez-López J. (2020). Damage Evolution Modeling for Steel Structures Subjected to Combined High Cycle Fatigue and High-Intensity Dynamic Loadings. Int. J. Struct. Stab. Dyn..

[24] Chen F., Jin Z., Wang E., Wang L., Jiang Y., Guo P., Gao X., He X. (2021). Relationship model between surface strain of concrete and expansion force of reinforcement rust. Sci. Rep..

[25] Chen F., Zhong Y., Gao X., Jin Z., Wang E., Zhu F., Shao X., He X. (2021). Non-uniform model of relationship between surface strain and rust expansion force of reinforced concrete. Sci. Rep..

[26] Mousavi A.A., Zhang C., Masri S.F., Gholipour G. (2022). Structural damage detection method based on the complete ensemble empirical mode decomposition with adaptive noise: A model steel truss bridge case study. Struct. Health Monit..

[27] Zhang S.S., Yu T., Chen G.M. (2017). Reinforced concrete beams strengthened in flexure with near-surface mounted (NSM) CFRP strips: Current status and research needs. Compos. Part. B Eng..

[28] Ghorbani M., Mostofinejad D., Hosseini A. (2017). Experimental investigation into bond behavior of FRP-to-concrete under mixed-mode I/II loading. Constr. Build. Mater..

[29] Bakay R., Shrive N.G., Sayed-Ahmed E.Y. (2009). Bond Strength of FRP Laminates to Concrete: State-of-the-Art Review. Electron. J. Struct. Eng..

[30] Xu J., Wu Z., Chen H., Shao L., Zhou X., Wang S. (2022). Influence of dry-wet cycles on the strength behavior of basalt-fiber reinforced loess. Eng. Geol..

[31] Wu Z., Xu J., Chen H., Shao L., Zhou X., Wang S. (2022). Shear Strength and Mesoscopic Characteristics of Basalt Fiber–Reinforced Loess after Dry–Wet Cycles. J. Mater. Civil. Eng..

[32] Huang H., Huang M., Zhang W., Pospisil S., Wu T. (2020). Experimental Investigation on Rehabilitation of Corroded RC Columns with BSP and HPFL under Combined Loadings. J. Struct. Eng..

[33] Wei J., Xie Z., Zhang W., Luo X., Yang Y., Chen B. (2021). Experimental study on circular steel tube-confined reinforced UHPC columns under axial loading. Eng. Struct..

[34] Teng J., Chen J.-F., Yu T. (2002). FRP-Strengthened RC Structures..

[35] Dai Minh Nguyen B., Khuan Chan T., Kiat Cheong H. (2001). Brittle Failure and Bond Development Length of Cfrp-Concrete Beams. J. Compos. Constr..

[36] Smith S.T., Teng J.G. (2002). FRP-strengthened RC beams. I: Review of debonding strength models. Eng. Struct..

[37] Lu Z., Xian G., Li H. (2015). Effects of exposure to elevated temperatures and subsequent immersion in water or alkaline solution on the mechanical properties of pultruded BFRP plates. Compos. Part. B Eng..

[38] Jiang C., Wan B., Wu Y.F., Omboko J. (2018). Epoxy interlocking: A novel approach to enhance FRP-to-concrete bond behavior. Constr. Build. Mater..

[39] Chajes M.J., Finch W.W., Januszka T.F., Thomson T.A. (1996). Bond and force transfer of composite material plates bonded to concrete. ACI Struct. J..

[40] Mostofinejad D., Mahmoudabadi E. (2010). Grooving as Alternative Method of Surface Preparation to Postpone Debonding of FRP Laminates in Concrete Beams. J. Compos. Constr..

[41] Nanni A. (2000). FRP Reinforcement for Bridge Structures. Proceedings, Strucural Engineering Conference.

[42] Jung W.T., Park J.S., Kang J.Y., Keum M.S. (2017). Flexural behavior of concrete beam strengthened by near-surface mounted CFRP reinforcement using equivalent section model. Adv. Mater. Sci. Eng..

[43] Bencardino F., Condello A., Ashour A.F. (2017). Single-lap shear bond tests on Steel Reinforced Geopolymeric Matrix-concrete joints. Compos. Part. B Eng..

[44] Heydari Mofrad M., Mostofinejad D., Hosseini A. (2019). A generic non-linear bond-slip model for CFRP composites bonded to concrete substrate using EBR and EBROG techniques. Compos. Struct..

[45] Al-Jaberi Z., Myers J.J., Chandrashekhara K. (2019). Effect of direct service temperature exposure on the bond behavior between advanced composites and CMU using NSM and EB techniques. Compos. Struct..

[46] Bardhan A., Samui P., Ghosh K., Gandomi A.H., Bhattacharyya S. (2021). ELM-based adaptive neuro swarm intelligence techniques for predicting the California bearing ratio of soils in soaked conditions. Appl. Soft Comput..

[47] Biswas R., Samui P., Rai B. (2019). Determination of compressive strength using relevance vector machine and emotional neural network. Asian J. Civil. Eng..

[48] Jalal F.E., Xu Y., Iqbal M., Jamhiri B., Javed M.F. (2021). Predicting the compaction characteristics of expansive soils using two genetic programming-based algorithms. Transp. Geotech..

[49] Kumar S., Rai B., Biswas R., Samui P., Kim D. (2020). Prediction of rapid chloride permeability of self-compacting concrete using Multivariate Adaptive Regression Spline and Minimax Probability Machine Regression. J. Build. Eng..

[50] Biswas R., Rai B. (2020). Effect of cementing efficiency factor on the mechanical properties of concrete incorporating silica fume. J. Struct. Integr. Maint..

[51] Biswas R., Rai B., Samui P., Roy S.S. (2020). Estimating concrete compressive strength using MARS, LSSVM and GP. Eng. J..

[52] Biswas R., Bardhan A., Samui P., Rai B., Nayak S., Armaghani D.J. (2021). Efficient soft computing techniques for the prediction of compressive strength of geopolymer concrete. Comput. Concr..

[53] Khan M.A., Memon S.A., Farooq F., Javed M.F., Aslam F., Alyousef R. (2021). Compressive Strength of Fly-Ash-Based Geopolymer Concrete by Gene Expression Programming and Random Forest. Adv. Civil. Eng..

[54] Khan M.I., Sutanto M.H., Khan K., Iqbal M., Napiah M., Zoorob S.E., Klemeš J.J., Bokhari A., Rafiq W. (2022). Effective use of recycled waste PET in cementitious grouts for developing sustainable semi-flexible pavement surfacing using artificial neural network (ANN). J. Clean. Prod..

[55] Iqbal M., Zhang D., Jalal F.E., Faisal Javed M. (2021). Computational AI prediction models for residual tensile strength of GFRP bars aged in the alkaline concrete environment. Ocean. Engineering.

[56] Iqbal M., Zhao Q., Zhang D., Jalal F.E., Jamal A. (2021). Evaluation of tensile strength degradation of GFRP rebars in harsh alkaline conditions using non-linear genetic-based models. Mater. Struct./Mater. Et Constr..

[57] Zhang D., Gu X.L., Yu Q.Q., Huang H., Wan B., Jiang C. (2018). Fully probabilistic analysis of FRP-to-concrete bonded joints considering model uncertainty. Compos. Struct..

[58] Huang H., Guo M., Zhang W., Huang M. (2022). Seismic Behavior of Strengthened RC Columns under Combined Loadings. J. Bridge Eng..

[59] Wang X., Yang Y., Yang R., Liu P. (2022). Experimental Analysis of Bearing Capacity of Basalt Fiber Reinforced Concrete Short Columns under Axial Compression. Coatings.

[60] Zhang W., Liu X., Huang Y. (2022). Reliability-based analysis of the flexural strength of concrete beams reinforced with hybrid BFRP and steel rebars. Archiv. Civ. Mech. Eng..

[61] Hu Z., Shi T., Cen M., Wang J., Zhao X., Zeng C., Zhou Y., Fan Y., Liu Y., Zhao Z. (2022). Research progress on lunar and Martian concrete. Constr. Build. Mater..

[62] Sun D., Huo J., Chen H., Dong Z., Ren R. (2022). Experimental study of fretting fatigue in dovetail assembly considering temperature effect based on damage mechanics method. Eng. Fail. Anal..

[63] Hao R.B., Lu Z.Q., Ding H., Chen L.Q. (2022). A nonlinear vibration isolator supported on a flexible plate: Analysis and experiment. Nonlinear Dyn..

[64] Zhang Z., Yang Q., Yu Z., Wang H., Zhang T. (2022). Influence of Y2o3 Addition on the Microstructure of Tic Reinforced Ti-Based Composite Coating Prepared by Laser Cladding. SSRN Electron. J..

[65] Liu S., Sai Q., Wang S., Williams J. (2022). Effects of Laser Surface Texturing and Lubrication on the Vibrational and Tribological Performance of Sliding Contact. Lubricants.

[66] Cheng H., Sun L., Wang Y., Chen X. (2021). Effects of actual loading waveforms on the fatigue behaviours of asphalt mixtures. Int. J. Fatigue.

[67] Cheng H., Liu L., Sun L. (2022). Bridging the gap between laboratory and field moduli of asphalt layer for pavement design and assessment: A comprehensive loading frequency-based approach. Front. Struct. Civil. Eng..

[68] Guo Y., Yang Y., Kong Z., He J. (2022). Development of Similar Materials for Liquid-Solid Coupling and Its Application in Water Outburst and Mud Outburst Model Test of Deep Tunnel. Geofluids.

[69] Shi L., Xiao X., Wang X., Liang H., Wang D. (2022). Mesostructural characteristics and evaluation of asphalt mixture contact chain complex networks. Constr. Build. Mater..

[70] Shi T., Liu Y., Zhang Y., Lan Y., Zhao Q., Zhao Y., Wang H. (2022). Calcined Attapulgite Clay as Supplementary Cementing Material: Thermal Treatment, Hydration Activity and Mechanical Properties. Int. J. Concr. Struct. Mater..

[71] Lan M.Y., Zheng M.B., Shi T., Ma C., Liu Y., Zhao Z. (2022). Crack resistance property of carbon nanotubes-modified concrete. Mag. Concr. Res..

[72] Prakash S., Kumar S., Biswas R., Rai B. (2022). Influence of silica fume and ground granulated blast furnace slag on the engineering properties of ultra-high-performance concrete. Innov. Infrastruct. Solut..

[73] Biswas R., Rai B. (2019). Efficiency Concepts and Models that Evaluates the Strength of Concretes Containing Different Supplementary Cementitious Materials. Civil. Eng. J..

[74] Bardhan A., Biswas R., Kardani N., Iqbal M., Samui P., Singh M.P., Asteris P.G. (2022). A novel integrated approach of augmented grey wolf optimizer and ANN for estimating axial load carrying-capacity of concrete-filled steel tube columns. Constr. Build. Mater..

[75] Biswas R., Rai B., Samui P. (2021). Compressive strength prediction model of high-strength concrete with silica fume by destructive and non-destructive technique. Innov. Infrastruct. Solut..

[76] Kumar M., Kumar V., Biswas R., Samui P., Kaloop M.R., Alzara M., Yosri A.M. (2022). Hybrid ELM and MARS-Based Prediction Model for Bearing Capacity of Shallow Foundation. Processes.

[77] Vu D.T., Hoang N.D. (2016). Punching shear capacity estimation of FRP-reinforced concrete slabs using a hybrid machine learning approach. Struct. Infrastruct. Eng..

[78] Hoang N.D. (2019). Estimating punching shear capacity of steel fibre reinforced concrete slabs using sequential piecewise multiple linear regression and artificial neural network. Meas. J. Int. Meas. Confed..

[79] Abuodeh O.R., Abdalla J.A., Hawileh R.A. (2020). Prediction of shear strength and behavior of RC beams strengthened with externally bonded FRP sheets using machine learning techniques. Compos. Struct..

[80] Su M., Zhong Q., Peng H., Li S. (2021). Selected machine learning approaches for predicting the interfacial bond strength between FRPs and concrete. Constr. Build. Mater..

[81] Kecman V. (2001). Learning and Soft Computing: Support Vector Machines, Neural Networks, and Fuzzy Logic Models.

[82] Lu M., AbouRizk S.M., Hermann U.H. (2001). Sensitivity Analysis of Neural Networks in Spool Fabrication Productivity Studies. J. Comput. Civil. Eng..

[83] Gupta A. (2021). Investigation of the strength of ground granulated blast furnace slag based geopolymer composite with silica fume. Mater. Today Proc..

[84] Holland J.H. (1975). Adaptation in Natural and Artificial Systems.

[85] Huang G.-B., Kheong Siew C., Zhu Q.-Y., Siew C.-K. Extreme learning machine: A new learning scheme of feedforward neural networks Sentence level sentiment analysis View project Neural Networks View project Extreme Learning Machine: A New Learning Scheme of Feedforward Neural Networks. Proceedings of the 2004 IEEE International Joint Conference on Neural Networks.

[86] Kardani N., Bardhan A., Kim D., Samui P., Zhou A. (2021). Modelling the energy performance of residential buildings using advanced computational frameworks based on RVM, GMDH, ANFIS-BBO and ANFIS-IPSO. J. Build. Eng..

[87] Friedman J.H. (1991). Multivariate Adaptive Regression Splines. Ann. Stat..

[88] Samui P. (2019). Application of Artificial Intelligence in Geo-Engineering. Springer Ser. Geomech. Geoengin..

[89] Bardhan A., Kardani N., GuhaRay A., Burman A., Samui P., Zhang Y. (2021). Hybrid ensemble soft computing approach for predicting penetration rate of tunnel boring machine in a rock environment. J. Rock Mech. Geotech. Eng..

[90] Asteris P.G., Skentou A.D., Bardhan A., Samui P., Pilakoutas K. (2021). Predicting concrete compressive strength using hybrid ensembling of surrogate machine learning models. Cem. Concr. Res..

[91] Liang Q.Q. (2009). Performance-based analysis of concrete-filled steel tubular beam-columns, Part I: Theory and algorithms. J. Constr. Steel Res..

[92] Vapnik V., Golowich S.E., Smola A. (1997). Support vector method for function approximation, regression estimation, and signal processing. Adv. Neural Inf. Processing Syst..

[93] Karimi Y., Prasher S.O., Patel R.M., Kim S.H. (2006). Application of support vector machine technology for weed and nitrogen stress detection in corn. Comput. Electron. Agric..

[94] Trebar M., Steele N. (2008). Application of distributed SVM architectures in classifying forest data cover types. Comput. Electron. Agric..

[95] Padmini D., Ilamparuthi K., Sudheer K.P. (2008). Ultimate bearing capacity prediction of shallow foundations on cohesionless soils using neurofuzzy models. Comput. Geotech..

[96] Kang F., Li J.-S., Wang Y., Li J. (2017). Extreme learning machine-based surrogate model for analyzing system reliability of soil slopes. Eur. J. Environ. Civil. Eng..

[97] Bardhan A., GuhaRay A., Gupta S., Pradhan B., Gokceoglu C. (2022). A novel integrated approach of ELM and modified equilibrium optimizer for predicting soil compression index of subgrade layer of Dedicated Freight Corridor. Transp. Geotech..

[98] Pradeep T., GuhaRay A., Bardhan A., Samui P., Kumar S., Armaghani D.J. (2022). Reliability and Prediction of Embedment Depth of Sheet pile Walls Using Hybrid ANN with Optimization Techniques. Arab. J. Sci. Eng..

[99] Ghani S., Kumari S., Bardhan A. (2021). A novel liquefaction study for fine-grained soil using PCA-based hybrid soft computing models. Sadhana–Acad. Proc. Eng. Sci..

[100] Kardani N., Bardhan A., Gupta S., Samui P., Nazem M., Zhang Y., Zhou A. (2021). Predicting permeability of tight carbonates using a hybrid machine learning approach of modified equilibrium optimizer and extreme learning machine. Acta Geotech..

[101] Bardhan A., Samui P. (2022). Application of Artificial Intelligence Techniques in Slope Stability Analysis: A Short Review and Future Prospects. Int. J. Geotech. Earthq. Eng..

[102] Pradeep T., Bardhan A., Burman A., Samui P. (2021). Rock Strain Prediction Using Deep Neural Network and Hybrid Models of ANFIS and Meta-Heuristic Optimization Algorithms. Infrastructures.

[103] Bardhan A., Kardani N., Alzoùbi A.K., Roy B., Samui P., Gandomi A.H. (2022). Novel integration of extreme learning machine and improved Harris hawks optimization with particle swarm optimization-based mutation for predicting soil consolidation parameter. J. Rock Mech. Geotech. Eng..

[104] Dhilipkumar B., Bardhan A., Samui P., Kumar S. (2021). Predicting Probability of Liquefaction Susceptibility Based on a Wide Range of CPT Data. Int. J. Geotech. Earthq. Eng..

[105] Bardhan A., Manna P., Kumar V., Burman A., Zlender B., Samui P. (2022). Reliability Analysis of Piled Raft Foundation Using a Novel Hybrid Approach of ANN and Equilibrium Optimizer. CMES Comput. Modeling Eng. Sci..

[106] Kardani N., Bardhan A., Samui P., Nazem M., Asteris P.G., Zhou A. (2022). Predicting the thermal conductivity of soils using integrated approach of ANN and PSO with adaptive and time-varying acceleration coefficients. Int. J. Therm. Sci..

[107] Kumar M., Bardhan A., Samui P., Hu J.W., Kaloop M.R. (2021). Reliability Analysis of Pile Foundation Using Soft Computing Techniques: A Comparative Study. Processes.

[108] Bardhan A., Gokceoglu C., Burman A., Samui P., Asteris P.G. (2021). Efficient computational techniques for predicting the California bearing ratio of soil in soaked conditions. Eng. Geol..

[109] Bardhan A., Kardani N., Alzo’ubi A.K., Samui P., Gandomi A.H., Gokceoglu C. (2022). A Comparative Analysis of Hybrid Computational Models Constructed with Swarm Intelligence Algorithms for Estimating Soil Compression Index. Arch. Comput. Methods Eng..

[110] Asteris P.G., Skentou A.D., Bardhan A., Samui P., Lourenço P.B. (2021). Soft computing techniques for the prediction of concrete compressive strength using Non-Destructive tests. Constr. Build. Mater..

[111] Biswas R., Li E., Zhang N., Kumar S., Rai B., Zhou J. (2022). Development of Hybrid Models Using Metaheuristic Optimization Techniques to Predict the Carbonation Depth of Fly Ash Concrete. SSRN Electron. J..

[112] Bardhan A., Samui P. (2022). Probabilistic slope stability analysis of Heavy-haul freight corridor using a hybrid machine learning paradigm. Transp. Geotech..

[113] Topal U., Goodarzimehr V., Bardhan A., Vo-Duy T., Shojaee S. (2022). Maximization of the fundamental frequency of the FG-CNTRC quadrilateral plates using a new hybrid PSOG algorithm. Compos. Struct..

[114] Amjad Raja M.N., Abbas Jaffar S.T., Bardhan A., Shukla S.K. (2022). Predicting and validating the load-settlement behavior of large-scale geosynthetic-reinforced soil abutments using hybrid intelligent modeling. J. Rock Mech. Geotech. Eng..

[115] Das G., Burman A., Bardhan A., Kumar S., Choudhary S.S., Samui P. (2022). Risk estimation of soil slope stability problems. Arab. J. Geosci..

[116] Chandra S., Agrawal S., Chauhan D.S. (2018). Soft computing based approach to evaluate the performance of solar PV module considering wind effect in laboratory condition. Energy Rep..

[117] Bhadana V., Jalal A.S., Pathak P. (2020). A comparative study of machine learning models for COVID-19 prediction in India. Proceedings of the 2020 IEEE 4th Conference on Information & Communication Technology (CICT).

[118] Pandey S.K., Janghel R.R., Mishra P.K., Kaabra R. (2022). Machine learning based COVID -19 disease recognition using CT images of SIRM database. J. Med. Eng. Technol..

[119] Rathor S., Jadon R.S. (2019). Acoustic domain classification and recognition through ensemble based multilevel classification. J. Ambient Intell. Humaniz. Comput..

[120] Kardani N., Bardhan A., Samui P., Nazem M., Zhou A., Armaghani D.J. (2021). A novel technique based on the improved firefly algorithm coupled with extreme learning machine (ELM-IFF) for predicting the thermal conductivity of soil. Eng. Comput..

